# Retrospective analysis of early neurodevelopmental outcomes after esophageal atresia repair at a single institution: *short-gap* vs. *long-gap* defect

**DOI:** 10.3389/fped.2025.1527880

**Published:** 2025-02-28

**Authors:** Mary Madelyn Lowdermilk, Devon Michael Evanovich, Jue Teresa Wang, Danielle Bennett Pier, Anjali Sadhwani, Benjamin Zendejas, Dusica Bajic

**Affiliations:** ^1^ Tufts University School of Medicine, Boston, MA, United States; ^2^ Department of Anesthesiology, Critical Care and Pain Medicine, Boston Children’s Hospital, Boston, MA, United States; ^3^ Harvard Medical School, Boston, MA, United States; ^4^ Department of Neurology, Division of Pediatric Neurology, Massachusetts General Hospital, Boston, MA, United States; ^5^ Department of Psychiatry and Behavioral Sciences, Boston Children’s Hospital, Boston, MA, United States; ^6^ Department of Surgery, Esophageal and Airway Treatment Center, Boston Children’s Hospital, Boston, MA, United States

**Keywords:** critical care, developmental delay, Foker process, neurology, prolonged sedation, pediatrics

## Abstract

**Background:**

With increased survival of infants born with esophageal atresia (EA), there is a knowledge gap regarding neurodevelopmental outcomes. We aimed to quantify the frequency of (1) documented developmental delay, and (2) implementation of early intervention services in the first and the second year of life following repair of *short-* and *long-gap* EA.

**Method:**

We retrospectively analyzed term-born (*n* = 44) and premature infants (*n* = 26) following EA repair at a single institution (2009–2020). Infants with anomalies associated with known neurological disorders were excluded. Clinical data was obtained from the electronic medical record, and presented as means and percentages. Developmental delay included clinically documented motor, speech/language, and cognitive delays that were stratified according to a surgical group: *short-* and *long-gap* EA.

**Results:**

Nearly half of *short-gap* (24/54; **44%**) and most of *long-gap* EA patients (12/16; **75%**) had documented developmental delay in the first year of life that persisted into the second year of life [**52%** [28/54] *short-gap*; **69%** [11/16] *long-gap* EA]. Developmental delay was noted irrespective of gestational age at birth, co-existing cardiac anomalies, or presence of cranial/brain findings on imaging. By age 2, **70%** (38/54) of *short-gap* and **69%** (11/16) of *long-gap* EA patients had received early intervention.

**Interpretation:**

Infants born with EA are at high-risk for developmental delay. Early neurodevelopmental assessments and intervention is recommended for EA patients.

## Highlights

• Evidence that esophageal atresia (EA) may be associated with impaired neurodevelopmental outcomes.
• Half of *short-gap* and most of *long-gap* EA had early developmental delay.
• Neurodevelopmental delay in EA is not associated with gestational age at birth.
• Most patients born with EA received early intervention by 2-years of age.
• Early implementation of baseline neurodevelopmental and brain imaging assessments should be considered.

## 1 Introduction

Esophageal atresia (EA), although a rare congenital anomaly, is one of the most common gastrointestinal birth defects (1) with a worldwide prevalence of 1 in 2,500 to 1 in 4,500 live births (2). In addition to anatomical classification in relation to presence and location of tracheoesophageal fistula(e) (*types A–D*), EA can also be classified as *short-gap* or *long-gap* EA based on the esophageal gap length which determines the duration and complexity of perioperative critical care (3). Infants born with a *long-gap* EA (gap length >3 cm or >2 vertebral bodies in length) (4) undergo multiple surgeries and more complex perioperative care. At our institution, repair of *long-gap* EA involves tension-induced esophageal growth over a period of several days to weeks as part of the staged Foker process (5, 6). The Foker process involves at least two separate thoracic operations to allow for esophageal growth using either external (5) or, more recently, internal traction (7). As a result, infants undergoing *long-gap* EA repair require prolonged sedation (5 days or more), which is known to be associated with development of tolerance and physical dependence (8). Similarly to infants born with *short-gap* EA, infants born with *long-gap* EA undergo serial follow-up esophagogastroduodenoscopies in the first year of life to evaluate and treat potential esophageal anastomotic strictures (9).

With increased survival of infants born with EA (10), there is a knowledge gap regarding neurodevelopmental outcomes. A recent systematic review of long-term neurodevelopment in children born with EA summarized conflicting results regarding outcomes (11), and the study did not specify the risk related to the surgical types of EA: *short-* vs. *long-gap* defect. Literature suggests that surgery for thoracic noncardiac congenital anomalies was associated with high-risk of brain injury leading to delay in neurocognitive and motor development (12), including those with EA (13, 14). Our recent pilot study highlighted potential risks of complex perioperative critical care as part of the Foker process on brain findings (15) and brain growth (16) following repair of *long-gap* EA.

The primary objective of the current retrospective study was to quantify the frequency of neurodevelopmental delay according to the complexity of perioperative care (*short*- vs. *long-gap* EA) in a recently described cohort of infants born with EA (10) that underwent repair at a single institution. We also investigated the frequency and timing of implementation of early intervention (EI) services. We hypothesized that early-to-late premature infants and those with *long-gap* esophageal atresia are at greater risk of developmental delay than those term-born and presenting with a *short-gap* EA.

## 2 Methods

### 2.1 Study design and participants

This study extends our previous analysis of a retrospective cohort of infants born with EA that underwent surgical repair at our institution from 2009 to 2020 (10). Institutional Review Board approved the study as a no greater than minimal risk. The study conformed to the standards set by the Declaration of Helsinki and Good Clinical Practice guidelines. Retrospective data were obtained from a prospectively maintained *Esophageal and Airway Treatment Center* REDCap database established in 2009. **Eligibility criteria** included: (1) term-born (37–42 weeks of gestation at birth), and (2) premature infants (28 to <37 weeks of gestation at birth (17) born with EA of any type that underwent surgical treatment at our institution. **Exclusion criteria** included (1) extreme prematurity [<28 weeks of gestation at birth (17)] as this group is already at high risk of neurodevelopmental findings (18), (2) any previous surgical intervention at other institutions, and (3) co-existing genetic or chromosomal abnormalities known to be associated with neurodevelopmental delays (*n* = 14).

Due to the retrospective study design, all data were obtained from the electronic medical record, Powerchart (Cerner, London, UK) as part of the clinical diagnostics and treatment. As previously described (10), we collected demographic and clinical characteristics including: (1) date of birth, (2) gestational age at birth (weeks), and (3) sex. Table 1 summarizes the characteristics of the retrospective cohort (*n* = 70) with respect to co-existing anomalies.

**Table 1:** Distribution of EA cohort with respect to co-anomalies.

EA study cohort characteristics	Number	Percentage (%)
I. EA as part of Syndrome	24/70	34%
VACTERL	24	34%
CHARGE	0	0
Other	0	0
II. EA with coexisting anomalies not part of Syndrome	46/70	66%
Anorectal	0	0%
Vertebral	9	20%
Cardiac	36	78%
Laryngeal Cleft	8	17%
Tracheo(broncho)malacia	23	50%
Limb	2	4%
Renal	12	26%

Table 1 Esophageal atresia (EA) cohort characteristics in the context of co-existing congenital anomalies. Our cohort included infants with EA as part of the VACTERL association (**34%**; 24/70). The table also lists co-existing anomalies for the remaining EA infants (**66%**; 46/70) who were not associated with a syndrome. Percentages for each subgroup were calculated within its respective group. Among those not part of the syndrome, the most common co-existing anomaly was a cardiac defect (**78%**; 36/46). We excluded 14 infants who had documented genetic or chromosomal abnormalities known to be associated with neurodevelopmental delays (*n* = 14; see Methods section). CHARGE, coloboma, heart defects, choanal atresia, growth retardation, genital abnormalities, and ear abnormalities; VACTERL, vertebral, anorectal, cardiac, tracheo-esophageal fistula and/ or esophageal atresia, renal, and limb defects/malformations.

#### 2.1.1 Surgical types of esophageal atresia

We categorized our retrospective cohort into *short-gap* (*n* = 54) and *long-gap* (*n* = 16) EA surgical groups. At our institution, the latter group undergo the Foker process (5, 6) that involves: (1) **Foker I** thoracotomy or thoracoscopy to place traction sutures onto the esophageal ends; (2) Time-on-traction with traction system adjustments; (3) **Foker II** thoracotomy or thoracoscopy to perform esophageal anastomosis; and (4) Postoperative sedation and subsequent weaning of sedation to allow for healing of the esophageal anastomosis (8, 16).

#### 2.1.2 Co-existing anomalies

Most patients in this retrospective analysis were born with co-existing cardiac anomalies (10) (Table 1). Due to the known increased risk of neurodevelopmental delays following cardiac repair (19, 20), co-existing cardiac anomalies were categorized into (1) major (requiring cardiac surgical repair), or (2) minor (not requiring surgical correction) as previously summarized [Table 2 in (10)]. Neurological findings of our cohort were obtained from clinically indicated cranial ultrasound and/or brain MRI findings [Table 2 in (21)]. Cranial imaging findings included the presence of abnormal head shape (e.g., plagiocephaly, brachycephaly, etc.), and/or signs of traumatic perinatal injury (e.g., cephalohematoma). Brain imaging findings ranged from likely benign (e.g., a simple cyst) to more serious findings [e.g., cerebral ventriculomegaly, brain atrophy, intracranial hemorrhage; see Figure 5 in (15)].

#### 2.1.3 Neurodevelopmental findings

In this retrospective review, our chart analysis focused on any mention of developmental delay, as documented in clinical notes from a variety of specialties, including developmental medicine, primary care, and physical therapy consultations. We categorized the frequency of neurodevelopmental outcomes in our cohort based on clinical documentation into three categories: (1) documented developmental delay, (2) documented normal development, and (3) not known—in cases when no clinical notes pertaining to neurodevelopmental assessment were available. The term “neurodevelopmental delay” refers to any type of neurodevelopmental impairment documented in the patients' clinical charts (e.g., motor delay, speech/language delay, cognitive delay, or failure to meet age-appropriate milestones) as recorded by a medical professional. Notably, feeding difficulties and/or dysphagia, which are common consequences of EA repair, were not considered a symptom of neurodevelopmental delay. We further categorized frequency of neurodevelopmental delay into three domains: (1) motor delay, (2) speech/language delay, and (3) cognitive delay, based on terminology used in the clinical notes. According to the clinicians’ reports, **motor delay** was characterized by abnormalities in gross-motor (e.g., large body complex movements and mobility) and/or fine-motor skills (e.g., visual-motor coordination, visual-spatial skills, and hand motor control). **Speech/language delay**, as noted in clinical reports, referred to delays in speech production, expressive language, and/or receptive language. **Cognitive delay** findings included memory, learning, and/or problem-solving delays as identified through neurodevelopmental assessments conducted by clinicians. Lastly, we collected the frequency of global developmental delay (GDD) as mentioned in the clinical notes. GDD refers to significant delays in two or more developmental domains (motor, language, cognitive, social interaction, and daily livings skills) (22).

#### 2.1.4 Documented developmental delay timing

We report the presence of neurodevelopmental delay documented in (i) the first year only, (ii) the second year only, or (iii) both the first and the second years of life. Additionally, we collected the earliest age (in months) of initial clinical documentation of any type of neurodevelopmental delay. For premature infants, we used corrected post-natal age that adjusted for gestational age at birth (in weeks) (23).

#### 2.1.5 Early intervention

At our institution, early intervention (EI) programs (24) that include a range of services and supports, are recommended for pediatric patients who show signs of developmental delays, as identified through neurodevelopmental assessments. Thus, we additionally retrospectively quantified the frequency of EI implementation by 2 years of age, based on clinical documentation. Additionally, we recorded the earliest age (corrected post-natal age in months) at which EI implementation was first documented.

### 2.2 Statistical analysis

Retrospectively collected data were presented as frequencies and percentages for documented neurodevelopmental delay and EI implementation in the context of (1) surgical classification of EA (*short-gap* vs. *long-gap* EA), (2) sex, (3) gestational age at birth (term-born vs. premature), and (4) co-existing cardiac anomalies and known cranial/brain findings. To enhance sample size and statistical power, frequency data were based on multiple assessments per patient and categorized for cross-sectional analysis at both the first and second years of life.

## 3 Results

**Figure 1:**
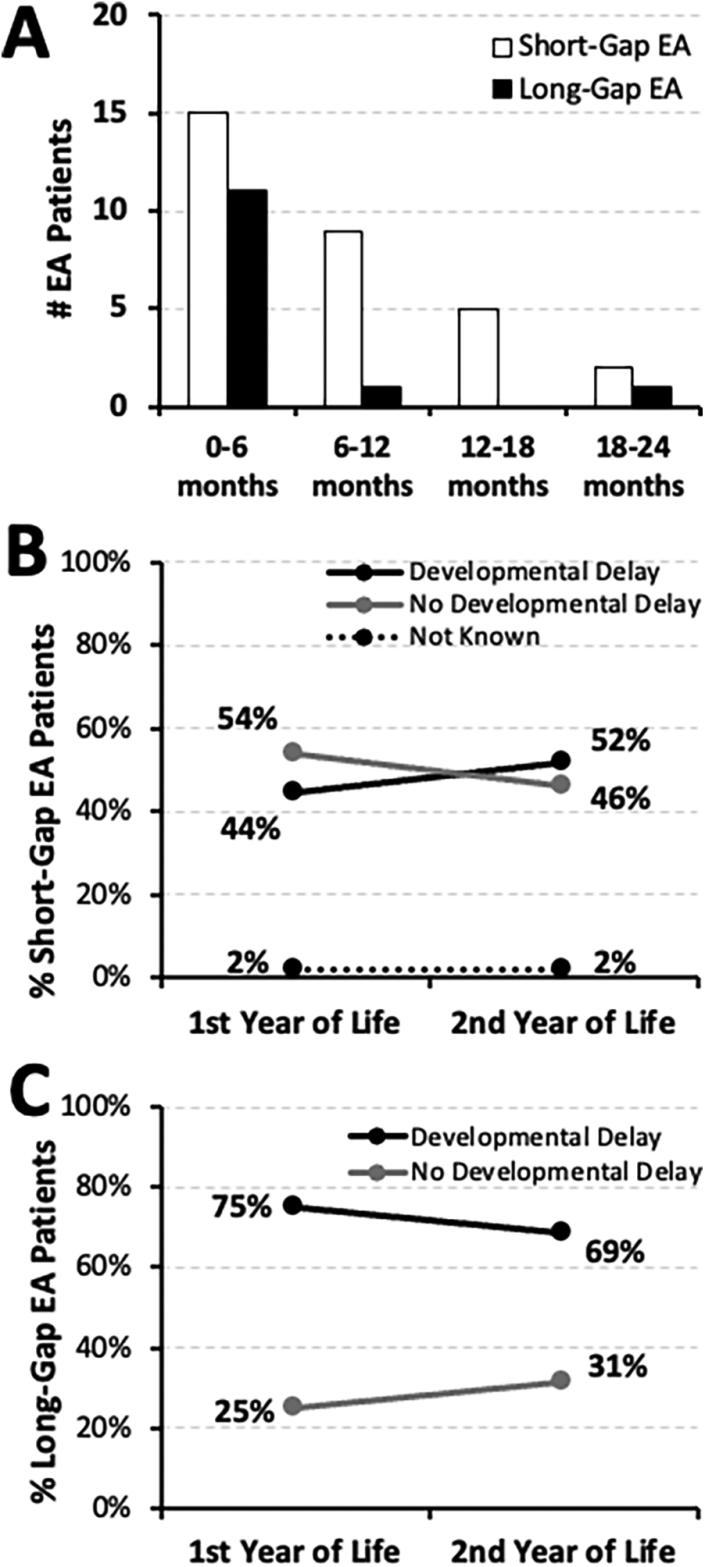
Earliest timing and frequency of documented developmental delay in infants born with esophageal atresia (EA). **(Panel A)** summarizes the absolute number (#) of infants with documented developmental delay across the first 2 years of life per earliest timing of clinical notes documentation (as corrected post-natal age in months): *short-gap* (*n* = 31/54; white bars), and *long-gap* EA (*n* = 13/16; black bars). This retrospective cohort includes infants that underwent EA repair at a single institution (2009-2020; *n* = 70) (10). Line graphs illustrate the change in occurrence of documented developmental delay between the two time points, the first and the second year of life, for infants born with *short-gap* (*n* = 54; **Panel B)** and *long-gap* EA (*n* = 16; **Panel C)**. Documented developmental delay data are shown as percent (%) and are classified as: present (black line), absent (gray line), or not known (dotted line).

We identified 54/70 (77%) *short-gap*, and 16/70 (23%) *long-gap* EA patients in our retrospective cohort (*n* = 44 term-born; *n* = 26 early-to-late premature) (10). Details of EA cohort (i) in the context of other congenital anomalies, and (ii) for surgical distribution of EA per sex, gestational age at birth, and gestational age at first EA surgery have been presented previously in Table 1 and Figure 1, respectively (21).

### 3.1 Neurodevelopmental findings in relation to demographic information

#### 3.1.1 Timing and frequency

The earliest documentation of developmental delay of any type was noted in the first 6 months of life (Figure 1A): **48%** (15/31) in the *short-gap* group, and **85%** (11/13) in the *long-gap* group. Indeed, neurodevelopmental delay was first documented over the course of the first year of life for most of the infants born with *short-gap* (24/31; **77%**) and *long-gap* EA (12/13; **92%**). For *short-gap* EA group (Figure 1B), nearly half had documented developmental delay in the first year (24/54; **44%**) and the second year of life (28/54; **52%**). For the *long-gap* EA group (Figure 1C), the majority had neurodevelopmental delay documented in the first (12/16; **75%**) and the second year of life (11/16; **69%**). The remainder of the cohort had documented normal neurodevelopment throughout the first two years of life, and only one *short-gap* EA patient lacked documentation regarding neurodevelopment (Figure 1B).

#### 3.1.2 Sex distribution

**Figure 2:**
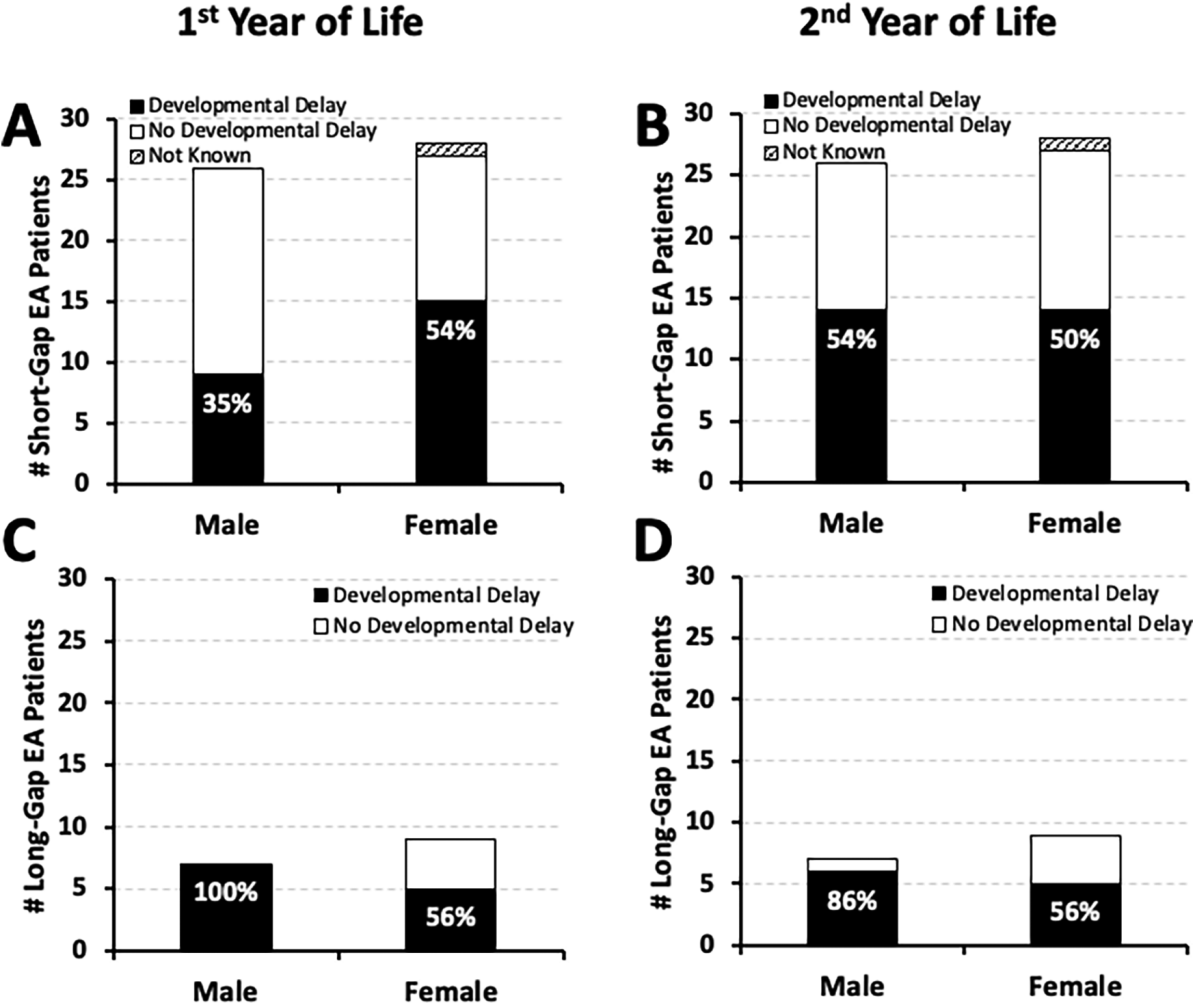
Frequency of documented developmental delay by Sex in infants born with esophageal atresia (EA). Cohort includes infants that underwent EA repair at a single institution from 2009 to 2020 (*n* = 70) (10). Graphs summarize the occurrence of documented developmental delay distributed by sex at two time points: the 1st year (left graphs), and the 2nd year of life (right graphs). Documented developmental delay data per sex are presented as absolute numbers (#) and are classified as: present (black bars), absent (white bars), or not known (stripes pattern bars) per male and female cohort infants born with *short-gap* (*n* = 54; **Panels A,B)** and *long-gap* EA (*n* = 16; **Panels C,D)**. Percentages (%) of developmental delay (black bars) are listed in each graph.

We report equal sex distribution of documented developmental delay by the second year of life: **50%** (14/28) male for *short-gap*, and **55%** (6/11) male for *long-gap* EA. Sex distribution for the remaining infants with no developmental delay and those with incomplete records is also illustrated in Figure 2.

#### 3.1.3 Gestational age at birth

**Figure 3:**
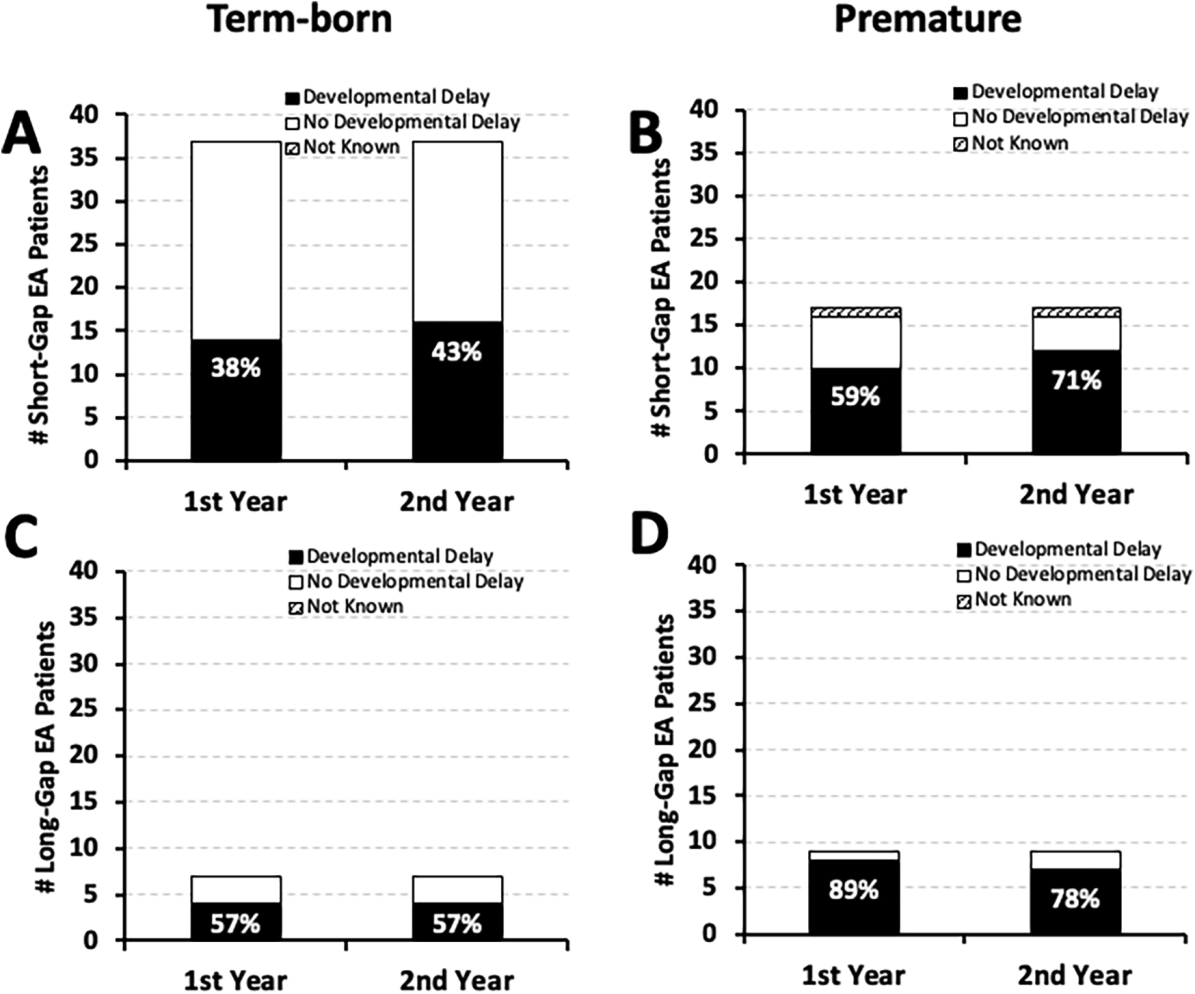
Frequency of documented developmental delay by gestational age at birth in infants born with esophageal atresia (EA). Graphs summarize the occurrence of documented developmental delay in the 1st and the 2nd year of life by gestational age in a retrospective cohort of infants that underwent EA repair at a single institution (2009–2020; *n* = 70) (10). The occurrence of documented developmental delay data are presented as absolute numbers (#), and are classified as: present (black bars), absent (white bars), or not known (stripes pattern bars) for term-born (37–42 weeks of gestation at birth; left column) and early-to-late premature infants (28 to <37 weeks of gestation of birth; right column) born with *short-gap* (*n* = 54; **Panels A,B)** and *long-gap* EA (*n* = 16; **Panels C,D)**. Percentages (%) of developmental delay (black bars) are listed in each graph.

As illustrated in Figure 3, our retrospective cohort (*n* = 70) has about two-thirds term-born (*n* *=* 44) and about one-third premature infants (*n* *=* 26). Nearly half of term-born infants with *short-gap* (16/37; **43%**) and *long-gap* EA (4/7; **57%**) had documented developmental delay by the second year of life (Figures 3A,C, respectively**)**. In contrast, the majority of premature patients had documented developmental delay regardless of the surgical type of EA: **71%** (12/17) *short-gap*, and **78%** (7/9) *long-gap* EA (Figures 3B,D). Interestingly, for *short-gap* EA infants with developmental delay in the first (*n* = 24/54) and the second year of life (*n* *=* 28/54), there is nearly equal distribution between term-born [**58**% [14/24] in the first; **57%** [16/28] in the second year of life] and premature groups. In contrast, *long-gap* EA patients with developmental delay (*n* = 12/16 in the first; *n* *=* 11/16 in the second year of life) were more frequently premature [**67**% [8/12] in the first; **64%** [7/11] in the second year of life] rather than term-born.

### 3.2 Neurodevelopmental findings in relation to co-existing anomalies

#### 3.2.1 Co-existing cardiac anomalies

**Figure 4:**
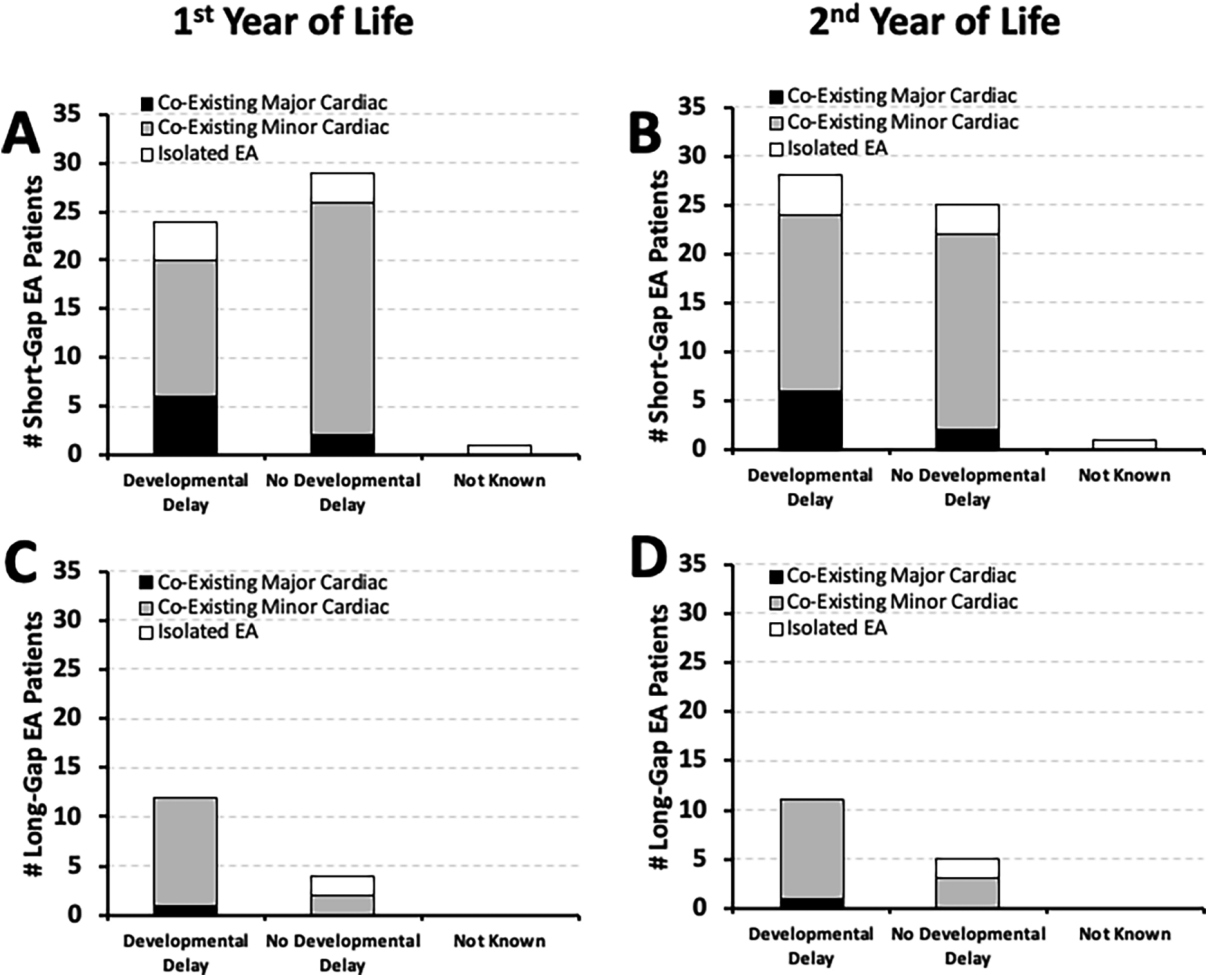
Frequency of documented developmental delay in relation to co-existing cardiac anomalies. Graphs show the incidence of documented developmental delay at two time points: the 1st year (0–12 months old; left graphs), and the 2nd year of life (12–24 months; right graphs) in relation to co-existing cardiac anomalies. The latter are described as either *minor* (gray bars) that did not require cardiac repair, or *major* cardiac anomalies (black bars) that required thoracic cardiac surgery. White bars denote isolated EA that did not have co-existing cardiac anomalies. Occurrence of documented developmental delay data are presented as absolute numbers (#) for two surgical groups: *short-gap* (*n* = 54; **Panels A,B)** and *long-gap* EA (*n* = 16; **Panels C,D)**.

It was previously reported [Table 2 in (10)] that most of the *short-gap* (38/54; **70%**) and *long-gap* EA patients (13/16; **81%**) in our cohort had minor co-existing cardiac anomalies that did not require surgical repair. Here, we report that the majority of *short-gap* (*n* = 28/54) and *long-gap* EA infants (*n* = 11/16) with documented developmental delay by the second year of life also had minor co-existing cardiac anomalies: **64%** (18/28) *short-gap* (Figures 4A,B), and **91%** (10/11) *long-gap* EA (Figures 4C,D). Interestingly, minor cardiac anomalies were also clinically noted in most of the infants with documented normal development in both surgical groups (Figure 4).

#### 3.2.2 Co-existing neurologic findings

**Figure 5:**
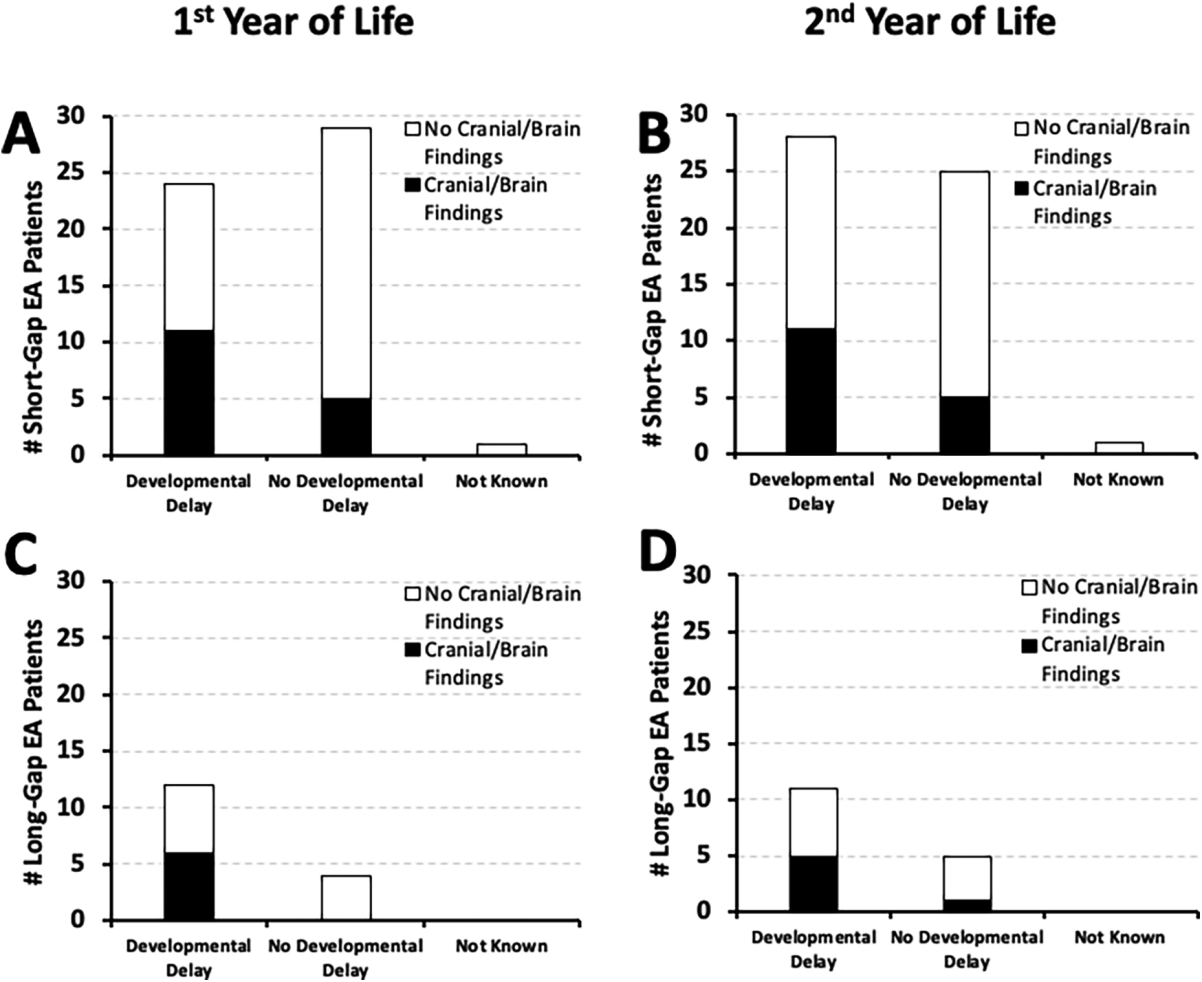
Frequency of documented developmental delay in relation to co-existing cranial/brain findings. Graphs show the occurrence of documented developmental delay at two time points: the 1st year (0–12 months old; left graphs), and the 2nd year of life (12–24 months old; right graphs) in relation to co-existing cranial/brain findings (10). Documented developmental delay data are presented as absolute numbers (#), and are classified as: present, absent, or not known per two surgical groups [*short-gap* EA, *n* = 54 [**Panels A,B**]; *long-gap* EA, *n* = 16 [**Panels C,D**]]. Presence of known co-existing cranial/brain findings in the 1st year of life is illustrated by black bars, while absence of co-existing findings or lack of information on possible cranial/brain findings is illustrated by white bars. Please refer to the text regarding the limitation of the underlying cranial/brain imaging diagnostics.

We previously summarized the type and frequency of neurologic diagnostics and neurological findings of the cohort [Table 2 in (18)]. Nearly half of *short-gap* EA patients with developmental delay (*n* = 24/56 in the first; *n* = 28/56 in the second year of life) had cranial/brain findings: **46%** (11/24; Figure 5A) in the first, and **39%** (11/28; Figure 5B) in the second year of life. Similar frequency of cranial/brain findings were also identified in *long-gap* EA patients with documented developmental delay (*n* = 12/16 in the first; *n* = 11/16 in the second year of life): **50%** (6/12; Figure 5C) in the first, and **45%** (5/11; Figure 5D) in the second year of life.

### 3.3 Timing and types of neurodevelopmental findings

**Figure 6:**
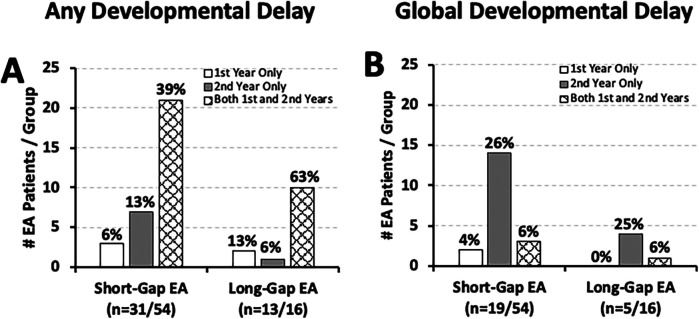
Timing of developmental delay and global developmental delay in esophageal atresia (EA) at a single institution. Graphs show the timing of developmental delay documentation in infants that underwent EA repair at a single institution (2009–2020; *n* = 70) (10) presented as: any type of developmental delay **(A)**, and global developmental delay **(B)**. See Methods section for definitions of developmental delay types. Graphs show data only for those with documented developmental delay per surgical group: *short-gap* (*n* = 31/54 in left graphs; *n* = 19/54 in right graphs), and *long-gap* EA (*n* *=* 13/16 in **A**; *n* = 5/16 in **B**). Graphs show absolute numbers (#) and percentage (%) per surgical group of each time-point.

Neurodevelopmental delay in the first two years of life was clinically documented in over half of *short-gap* (31/54; **57%**) and most of *long-gap* EA patients (13/16; **81%**). Indeed, as summarized in Figure 6A, documented neurodevelopmental delay “of any type” was identified in most of the EA patients in the first year of life and persisted into the second year of life irrespective of surgical type: **68%** (21/31) of *short-gap*, and **77%** (10/13) of *long-gap* EA. Only a few *short-gap* (3/31; **10%**) and *long-gap* EA patients (2/13; **15%**) had documented developmental delay in the first year that resolved by the second year of life. With respect to global developmental delay (GDD), about **35%** (19/54) of *short-gap* and **31%** (5/16) of *long-gap* EA patients had documented GDD only in the first 2 years of life (Figure 6B). Of those, clinical documentation of GDD was made primarily in the second year of life: *short-gap* (14/19; **74%**) and *long-gap* EA patients (4/5; **80%**).

**Figure 7:**
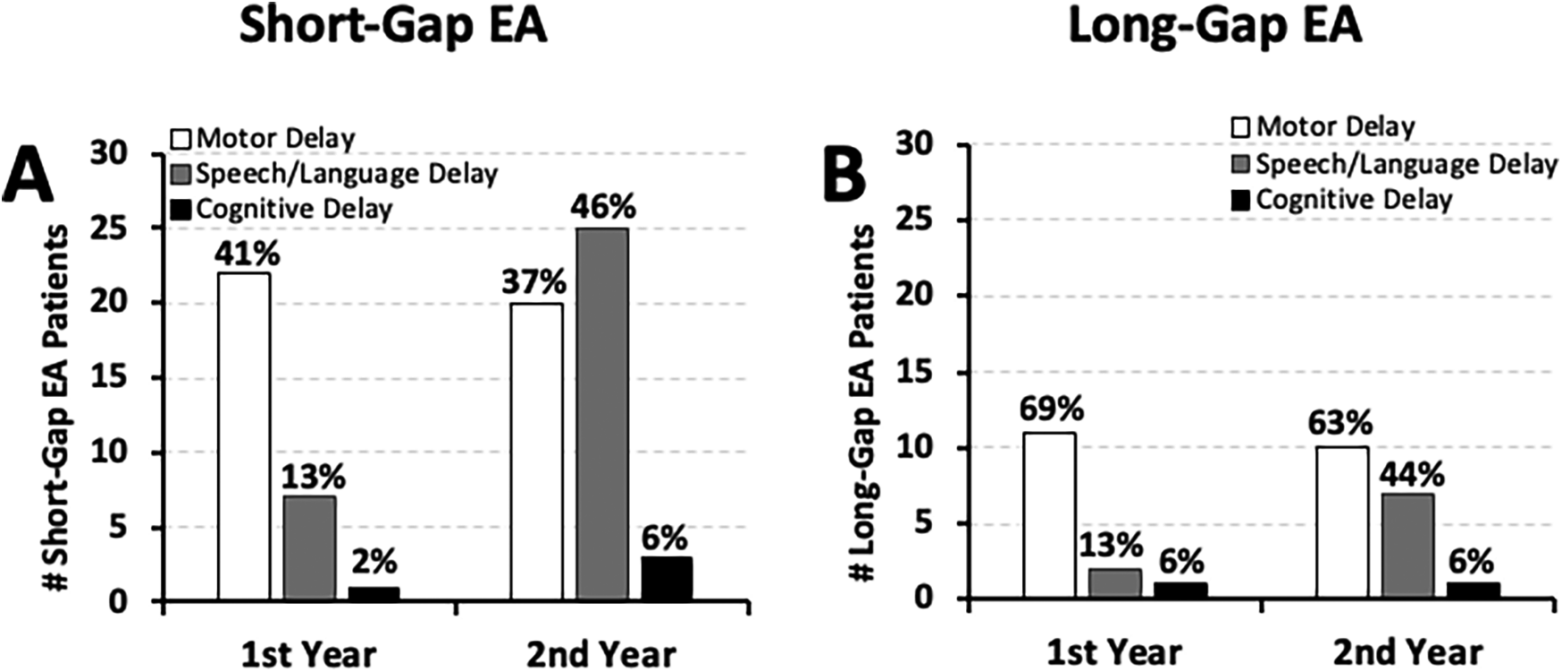
Frequency of documented developmental delay types in esophageal atresia (EA) at a single institution. Graphs summarize the occurrence of documented developmental delay types at two time points: the 1st year (0–12 months old), and the 2nd year of life (12–24 months old). Retrospectively collected measures include documented **(i)** motor (white bars), **(ii)** speech/language (gray bars), and **(iii)** cognitive delay (black bars) as per clinical notes for cohort infants born with *short-gap* (*n* = 54/70; **A**) and *long-gap* EA (*n* = 16/70; **B**). See Methods section for definitions of developmental delay types. Graphs show absolute numbers (#) and percentage (%) per surgical group of each time-point.

Specifically, Figure 7 illustrates that the earliest motor function delay was noted in the first year of life in both *short-gap* (22/54; **41%**) and *long-gap* EA patients (11/16; **69%**), and remained roughly the same in the second year of life [**37%** [20/54] *short-gap*; **63%** [10/16] *long-gap* EA]. As language typically develops in the second year of life, we accordingly report nearly half of the patients with documented delayed speech/language development: **46%** (25/54) *short-gap*, and **44%** (7/16) *long-gap* EA patients. Data for cognitive development should be interpreted with caution considering the sources of evaluation included physicians (non-psychologist) lacking formal training in cognitive assessment of young children.

### 3.4 Earliest timing and frequency of early intervention

**Figure 8:**
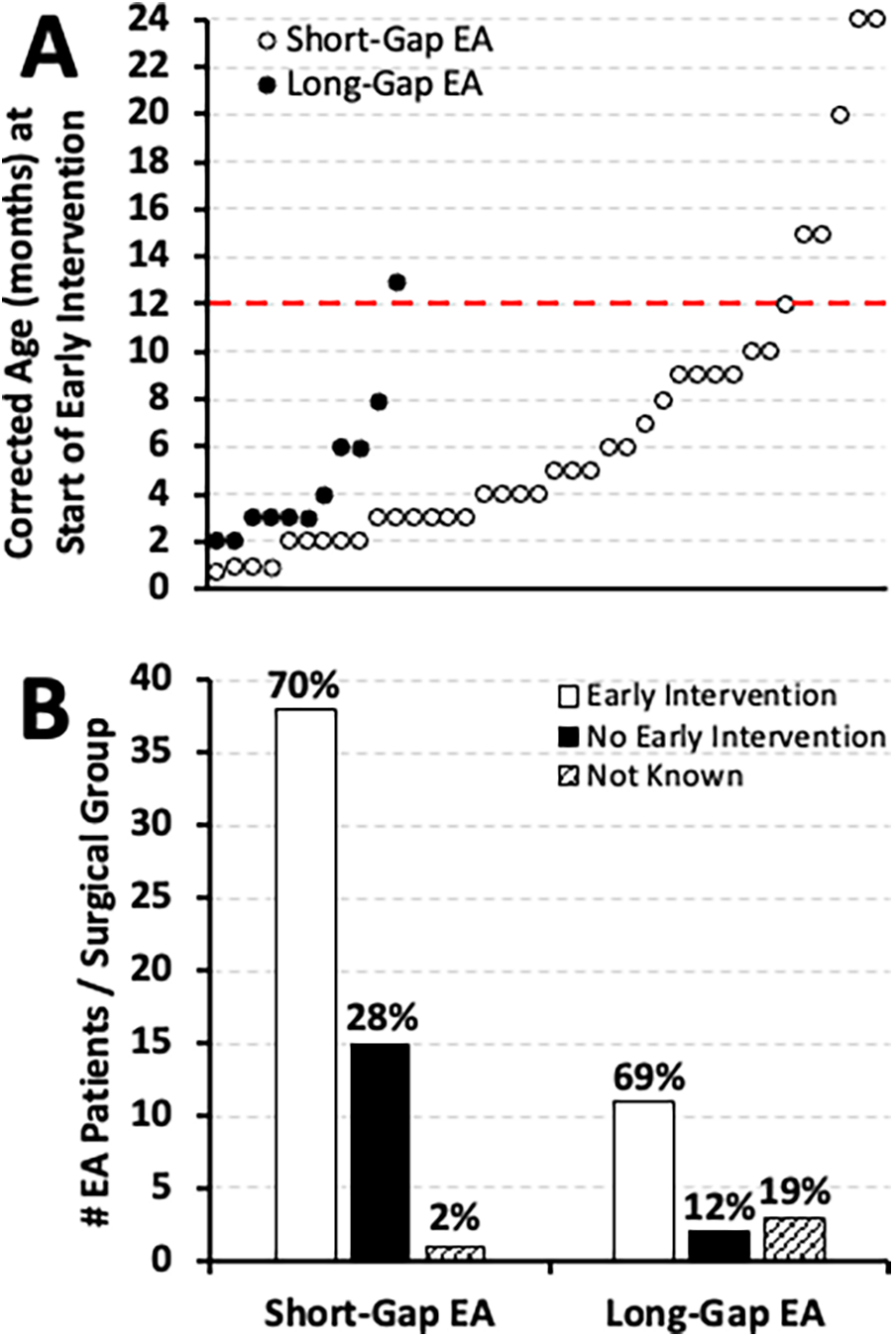
Early intervention implementation in infants that underwent esophageal atresia (EA) repair at a single institution. **(Panel A)** illustrates the individual timing of early intervention implementation by corrected age (in months) for two different surgical groups: *short-gap* (*n* = 54; white dots), and *long-gap* EA (*n* = 16; black dots). In contrast, **(Panel B)** shows the cohort frequency (#) of early intervention implementation by 2 years of age distributed by surgical group in absolute numbers while percentages (%) are summarized per surgical group. As described in Methods section, infants were enrolled in early intervention services either for existing developmental delay or for being at risk of developmental delay.

In our cohort, EI enrollment was noted at or before six months old for **63%** (24/38) of *short-gap* and **81%** (9/11) of *long-gap* EA patients and continued to be implemented across the first two years of life (see Figure 8A for individual distribution across time). Importantly, we report that **70%** (38/54) of *short-gap* and **69%** (11/16) of *long-gap* EA patients, were enrolled in EI services by two years of age (Figure 8B). Of the infants that underwent EI evaluation, developmental delay was clinically noted in **74%** (28/38) of *short-gap* and **91%** (10/11) of *long-gap* EA patients.

## 4 Discussion

Our retrospective analysis shows high occurrence of neurodevelopmental delay following both *short-gap* and *long-gap* EA repair, documented as early as the first six months of life. Clinical documentation of neurodevelopmental delay was noted irrespective of (1) gestational age at birth (term-born vs. premature), (2) co-existing cardiac anomalies, or (3) presence of cranial/brain imaging findings. Lastly, we report high frequency of EI implementation before two years of age in both *short-gap* and *long-gap* EA patients.

### 4.1 Occurrence of neurodevelopmental delay

#### 4.1.1 Timing of documented developmental delay

Our retrospective data show that infants born with EA, irrespective of the surgical type, are at risk of developmental delay throughout the first and the second year of life, documented as early as the first six months of life. Similarly, a study by Francesca et al. (25) reported development delays at 6 and 12 months, with lower average developmental scores at 12 months compared to 6 months. Another study by Walker et al. (26) reported developmental delay at 12 months of age in 52% of their cohort of EA patients. Neurodevelopmental delay in patients born with EA was also reported between 12 and 36 months in yet another study by Mawlana et al. (27). Neurodevelopmental delay was also noted as early as 12 months of age in other cohorts of infants that underwent complex critical care following repair of non-cardiac congenital anomalies (13), cardiac anomalies (20), and congenital diaphragmatic hernias (28).

#### 4.1.2 Frequency of documented developmental delay

In addition to high occurrence of documented developmental delay in *long-gap* EA patients, nearly half of infants born with *short-gap* EA in our retrospective cohort had developmental delay documented in the first two years of life. This finding suggests risk of early developmental delay irrespective of the complexity of surgical repair. Literature suggests that surgery for thoracic noncardiac congenital anomalies was associated with high-risk of brain injury (14–16) possibly leading to delay in neurocognitive and motor development (12), including those with EA (13). Given that infants with congenital gastrointestinal anomalies experience multiple stressors early in life, and the fact of improved survival rates of infants born with EA over the recent decade (10), prospective follow up studies are needed to comprehensively examine the impact of complex perioperative critical care on long-term neurodevelopmental outcomes in infants born with EA irrespective of the surgical type (*short-gap* and *long-gap* EA).

#### 4.1.3 Types of documented neurodevelopmental delay

We report findings of documented motor delay in both *short-* and *long-gap* EA groups in the first and the second year of life. This finding is consistent with the most recent reports of motor delays throughout the first two years of life in group of infants following EA repair (25, 26). Early assessment and detection of motor delays before 12 months old is important, as fine and gross motor functions such as grasping, sitting independently, and crawling typically appear during this timeframe. Assessment in the second year is similarly important as delays in gross motor functions such as standing and walking typically emerge between 12 and 15 months old (29). Nearly half of our cohort patients had also documented speech/language delays in the second year of life (Figure 7), which is when expressive and receptive language delays become apparent (29). A study by Mawlana et al. (27) similarly reported evidence of speech/language delays during this timeframe. As evaluation of cognitive functions are difficult to interpret before age two by non-psychologists lacking in specialized cognitive assessment expertise, our data regarding cognitive delays should be interpreted with caution. Previous reports by other groups also failed to detect cognitive developmental delay at this early age (9, 25, 26). Lastly, only a minority of *short-gap* and *long-gap* EA patients had documented GDD (Figure 6B). However, the frequency of GDD increased from the first to the second year of life, which in the context of GDD definition (see Methods), reflects increased detection of multiple types of developmental delays over time. Future studies in infants born with EA should involve administered neurobehavioral assessments (e.g., *Bayley Scales of Infant and Toddler Development*) at several time points in the first 3 years of life.

### 4.2 Neurodevelopmental findings in relation to possible confounders

#### 4.2.1 Sex distribution

Equal sex distribution of the cohort (10) and nearly equal sex distribution with respect to the frequency of documented developmental delay per surgical type (Figure 2) implies that there is no sex-specific vulnerability to developmental delay in infants born with EA. As such, future studies could potentially combine sex data with a goal of increasing the study power.

#### 4.2.2 Gestational age at birth

As it is well known that prematurity is a risk factor for neurodevelopmental delay (30), it is not surprising that we report higher frequency of early documented neurodevelopmental delay in premature infants (born 28 to <37 weeks of gestation) compared to term-born infants irrespective of the surgical group of EA (Figure 3). Our report highlights that the frequency of neurodevelopmental delay in term-born patients is higher than that in the general population. As such, our study represents a “call to action” to address the gap in our knowledge regarding the underlying mechanisms that lead to neurodevelopmental vulnerability of term-born infants following EA repair.

#### 4.2.3 Co-existing congenital cardiac anomalies

It is well known that infants are at risk for adverse neurodevelopmental outcomes following neonatal cardiac surgery (19, 20). As EA often presents with other congenital anomalies that can impact complexity of care (10, 21), the frequency of neurodevelopmental outcomes in relation to co-existing cardiac anomalies represent novel findings. We identified that most EA patients with documented early neurodevelopmental delay had only co-existing minor cardiac anomalies that did not require surgical repair (Figure 4).

#### 4.2.4 Presence of imaging findings

Documented neurodevelopmental delay was found both in the presence and absence of known cranial/brain findings irrespective of the surgical group (Figure 5). As we previously reported (21), the frequency of cranial/brain findings in our group (67% of those imaged; 22/33) may be underestimated, as neurologic diagnostic imaging was performed in only about half of the cohort (47%, 33/70). A study by Stolwijk et al. (14) reported that infants born with noncardiac congenital anomalies are at risk of postoperative brain injury following neonatal surgery, potentially contributing to the increased frequency of observed developmental delay in this population (11–13). Furthermore, our recent pilot MRI study following *long-gap* EA repair with Foker process reported clinically significant incidental imaging findings (15, 16) and reduction of brain volumes (15, 31, 32). Although EA is not known to be associated with neurological imaging findings or specific neurological sequelae (2), future prospective research is warranted to evaluate neurologic vulnerability and its impact on the risk of long-term neurodevelopmental sequelae.

### 4.3 Early intervention enrollment

In this report, we show EI implementation in most infants with EA irrespective of the surgical stratification (Figure 8). Similar frequency of EI enrollment was found in a cohort of infants undergoing surgical repair of congenital cardiac anomalies who were also at risk of developmental delay (20). Infants born with congenital cardiac anomalies are typically enrolled in EI programs initiated as early as the first month of life due to the high risk of neurodevelopmental delays (19). Thus, our novel findings highlight the need for timely detection with regular follow-up neurodevelopmental assessments regardless of surgical type, gestational age, or co-existing anomalies of infants born with EA. The more detailed aspects of the EI programs, including but not limited to *individualized* therapeutic services (e.g., physical therapy, occupational therapy, speech therapy, behavioral and cognitive developmental therapy), family service plans, and educational plans, are beyond the scope of this manuscript's analysis.

### 4.4 Limitations of the retrospective chart review

In the context of retrospective studies, data obtained from the medical records were originally intended for clinical use alone. Thus, the retrospective study design may lead to incomplete or missing documentation (e.g., unknown status of neurodevelopmental assessment and EI implementation). Despite our exclusion criteria, this study retained a moderate sample size with enough power to quantify early neurodevelopmental outcomes. The sample size *n* = 16 for infants born with *long-gap* EA is considered sufficient by literature to obtain both accurate and clinically relevant results (33).

## 5 Conclusion

This original retrospective analysis assessed the neurodevelopmental outcomes in a *novel* manner per surgical group: *short*- vs. *long-gap* EA. We report a high occurrence of documented neurodevelopmental delay as early as the first six months of life, regardless of gestational age at birth, surgical type, or co-existing anomalies, as well as a high frequency of EI enrollment. Our data is in support of early and regular neurodevelopmental assessments, EI enrolment, and long-term neurodevelopment follow-up programs of infants born with EA.

## Data Availability

The raw data supporting the conclusion of this article will be made available by the authors upon reasonable request.
